# Zebrafish as a Useful Model to Study Oxidative Stress-Linked Disorders: Focus on Flavonoids

**DOI:** 10.3390/antiox10050668

**Published:** 2021-04-25

**Authors:** Francesco Abbate, Alessandro Maugeri, Rosaria Laurà, Maria Levanti, Michele Navarra, Santa Cirmi, Antonino Germanà

**Affiliations:** 1 Department of Veterinary Sciences, University of Messina, 98168 Messina, Italy; rosaria.laura@unime.it (R.L.); maria.levanti@unime.it (M.L.); antonino.germana@unime.it (A.G.); 2 Department of Chemical, Biological, Pharmaceutical and Environmental Sciences, University of Messina, 98168 Messina, Italy; amaugeri@unime.it (A.M.); mnavarra@unime.it (M.N.); 3 Department of Pharmacy-Drug Sciences, University of Bari “Aldo Moro”, 70125 Bari, Italy

**Keywords:** zebrafish, flavonoids, oxidative stress, reactive oxygen species, inflammation, cancer, angiogenesis, ultraviolet radiation

## Abstract

The zebrafish is considered one of the most versatile experimental animal models. The transparency of the embryos, the small size, the rapid development and the homology with higher vertebrates have made the zebrafish a valuable model also for drug screening. Its use is closely related for the determination of bioactivity, toxicity and off-target side effects of novel drug candidates, which also allows a thorough evaluation of new targets; thus, it may represent a suitable model for drug screening and the optimization of novel candidates. Flavonoids are polyphenolic compounds widely present in fruits, vegetables and cereals. Polyphenols are important for both plants and humans, considering their involvement in defense mechanisms, particularly against oxidative stress. They protect plants from biotic and abiotic stressors and prevent or treat oxidative-based human diseases. For these reasons, polyphenols are used as nutraceuticals, functional foods and supplements by the pharmaceutical industry. Therefore, the most relevant findings on zebrafish as a useful experimental model to study oxidative stress-linked disorders, focusing on the biological activities of flavonoids, are here summarized and reviewed.

## 1. Introduction

### 1.1. Zebrafish as Experimental Model

The zebrafish, a small teleost species, has rapidly become a worldwide fundamental animal model in many scientific branches such as development analysis, molecular biology, genetics, immunity and for the study of several diseases [1,2]. Due to its small size, transparency of the embryos and rapid development, zebrafish has also become a relevant model for drug screening [3,4]. The main benefit of using zebrafish is that new potential drug candidates may be examined in a whole organism in a relatively shorter time and in a more economically advantageous model than rodents or other laboratory animals. Furthermore, the potentiality of zebrafish to be a model for a wide variety of human diseases is due to the high anatomical and physiological homology to humans [5]. The zebrafish was primarily used as a model to study embryology for its high fertility, external fertilization and embryo transparency. Moreover, the possibility to perform “knock-down” genetic ablations using the so-called “morpholino”, capable of blocking the translation of the messenger RNA, was extremely interesting. The embryo-larval zebrafish has also been used as xenograft model, in which human cancer cells grow, divide, metastasize and induce angiogenesis likewise in rodent xenograft models, with the significant advantage that fewer cancer cells are necessary for xenotransplantation and better simulation of earlier stages of cancer progression, hence providing a tool for identification of anti-invasive and anti-proliferative therapeutics in one assay [6,7].

**Figure 1:**
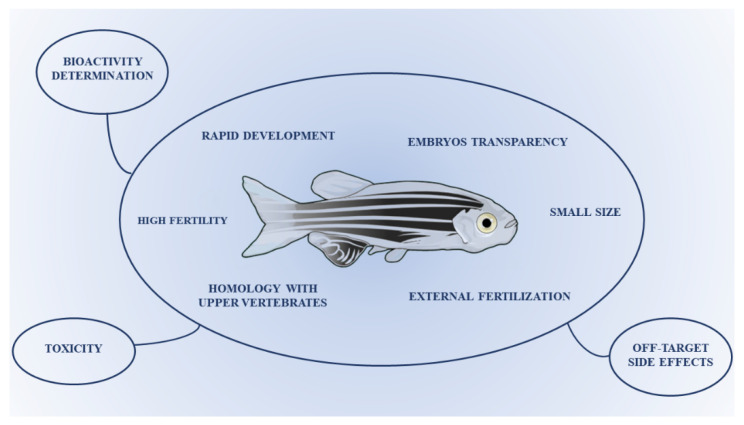
Characteristics of the zebrafish as a valuable model for drug screening and optimization.

Therefore, zebrafish is a valuable in vivo model, among the many successfully employed in preclinical studies, for the screening, optimization and bioactivity determination of lead compounds, also allowing the validation of novel drug targets [2,8,9] (Figure 1). Moreover, by means of the zebrafish model, the evaluation of drug toxicity and off-target side effects can be also carried out, being a significant pre-filter for the earliest choice of the safest drugs during their discovery process [5] (Figure 1). Overall, the possibility of assessing the effectiveness of several therapies (i.e., anti-cancer and neuroprotective), coupled with the abovementioned benefits of zebrafish (i.e., homology to upper vertebrates, ease of genetic modifications and advantageous phenotypical characteristics), offers real proof of the appropriateness of this experimental model in translational research in the optics of personalized medicine to move knowledge advancements from bench to clinic [10,11].

### 1.2. Flavonoids

**Figure 2:**
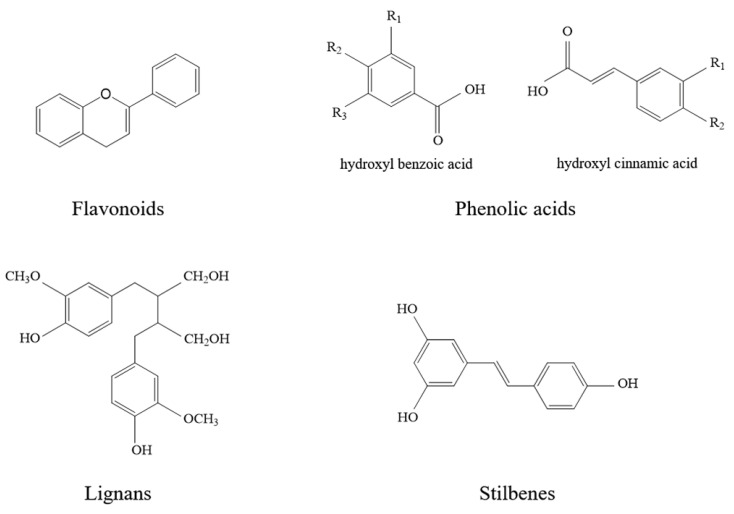
Chemical structures of the polyphenol classes.

Flavonoids are polyphenolic compounds largely present in vegetables, fruits and cereals. Many of the secondary metabolites are responsible for: (i) fruits, flowers and leaves colors; (ii) the organoleptic and qualitative properties of foods originating from edible fruits and plants; (iii) their juice astringency and bitterness. Polyphenols are involved in both plants and humans defense mechanisms, particularly against oxidative stress [12]. They protect plants from biotic and abiotic stress factors, along with being useful for preventing or treating certain oxidative-based human pathologies. Therefore, polyphenols are present in nutraceuticals, functional foods and supplements along with being widely exploited by the pharmaceutical industry. More than 8000 polyphenolic compounds have been identified, which are biosynthesized from phenylalanine via the shikimic acid pathway. The common scaffold is a benzene ring bearing one or more hydroxyl groups. The variety of structures are classified as a function of the number of phenol rings and on the basis of structural elements that bind these rings to one another. Polyphenols may be classified into flavonoids, phenolic acids, lignans and stilbenes (Figure 2).

**Figure 3:**
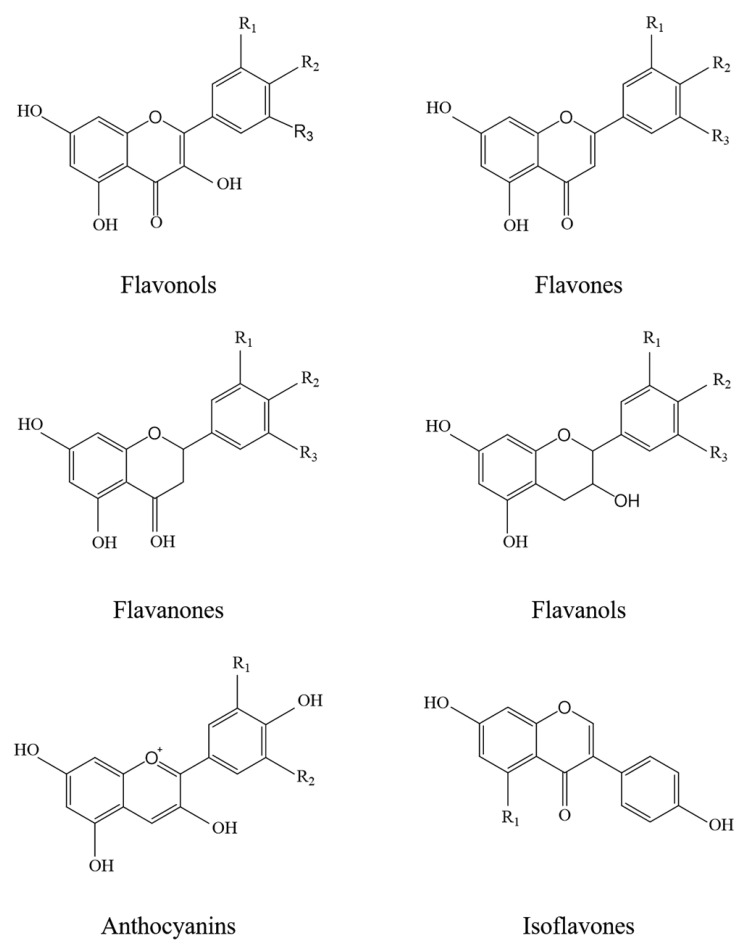
Chemical structures of the main subclasses of flavonoids.

Flavonoids are the most abundant polyphenols in our diet and the most studied ones. They are characterized by a common benzo-γ-pyrone moiety, variously substituted with hydroxyl and methoxyl groups. Based on their chemical structures, flavonoids may be divided into six major subclasses: flavonols, flavones, flavanones, flavanols, anthocyanins and isoflavones (Figure 3).

Their main dietary sources are citrus fruits, tea and red wine, among others. Numerous studies have suggested that regular intake of polyphenol-rich foods may have beneficial effects against a large spectrum of human pathologies including inflammatory-based, cardiovascular and neurodegenerative diseases, cancer, allergies, bacterial and viral infections, osteoporosis and diabetes [13,14,15,16,17], hence they could also be clinically exploited [18,19]. The capability of these natural compounds to improve health status was attributed especially to their antioxidant activity, being able to prevent and scavenge the formation of reactive oxygen species (ROS) and reactive nitrogen species (RNS), representing important hallmarks of inflammation [20,21,22]. Furthermore, they can influence cellular function by direct interaction with several receptors, as well as modulate intracellular signaling and transcription of gene involved in different pro-inflammatory pathways [23,24]. Flavonoids act as antioxidants through different mechanisms: (i) by scavenging free radicals; (ii) by chelating metallic ions that in turn could have catalyzed the generation of free radicals; (iii) by the donation of a proton from the phenolic compounds to the radicals; (iv) by inhibiting pro-oxidant enzymes, such as lipoxygenases, cyclooxygenases and xanthine oxidases, that generate free radicals; (v) by interfering with the oxidative/antioxidative potential of the cell [25]. Flavonoids influence cellular function also by direct interaction with both genes and proteins, thus modulating key signaling pathways linked to chronic degenerative diseases such as inflammation and cancer [26,27]. For instance, several reports documented the ability of flavonoids to: (i) inhibit redox-sensitive transcription factors, such as nuclear factor kappa B (NF-κB) and mitogen activated protein kinases (MAPK) superfamily [28,29]; (ii) suppress cyclooxygenase-2 (COX-2) and inducible nitric oxide synthase (iNOS) expression [30]; (iii) modulate the expression of B-cell lymphoma 2 (Bcl-2) family protein [31,32]; (iv) interfere with metalloproteinase and other adhesion molecules [33,34].

## 2. Effects of Flavonoids Employing Zebrafish as Experimental Model

### 2.1. Antioxidant Effects

It is known that excessive ROS production causes oxidative stress, especially damaging the liver and the brain [35]. In this regard, Tseng et al. in 2012 [36] studied the protection provided by quercetin against copper-induced oxidative stress. The anti-tumor, anti-inflammatory and anti-angiogenic activities of quercetin were previously described [37], as well as its antioxidant properties [38]. In cultured FL83B mouse hepatocytes, Cu^2+^ induced apoptosis increasing ROS levels. In transgenic zebrafish larvae, bearing a green fluorescent liver fatty acid-binding protein 1 (LFABP1:GFP), Cu^2+^ was already lethal at 10 μM after 4 days of exposure. The pretreatment with 15 μM of quercetin 3-O-methyl ether (Q3), a derivative commonly present in plants, significantly reduced the observed lethality, confirming its value in copper-induced toxicity. Moreover, the significant decrease in LFABP1 expression seen after the exposure to 5 μM Cu^2+^ was reversed in zebrafish cotreated with Q3. The same outcome was observed in both zebrafish adults and larvae, thus demonstrating the role of Q3 in the protection against oxidative damage in zebrafish hepatocytes, regardless the development stage.

Chen et al., in 2012 [39] used zebrafish larvae to evaluate the antioxidant activity of fifteen commercially available flavonoids against UV-induced phototoxicity, along with the computational quantitative structure–activity relationships (QSAR) method to investigate the correlations between the observed biological activities and the physico-chemical properties of the different compounds. Among these compounds, chrysin and morin showed higher ROS-scavenging rates (99% and 101%, respectively) and lower toxicity (LD50 > 100 ppm). In addition, zebrafish treated with chrysin and morin before UV exposure presented fins and organs particularly sensitive to UV radiation, respectively, 6.30- and 11.9-times more likely to grow to normal size than those in the UVB-only control group. Analyzing the results with QSAR method the, authors highlighted the relevance of the position of hydroxyl and amino groups within the flavonoid scaffold with the observed antioxidant effects. Therefore, the significance and value of the combination of QSAR method with zebrafish as an experimental model for the evaluation of flavonoids were clearly demonstrated.

In 2014, the effects of eriocitrin against the diet-induced hepatic steatosis by the activation of mitochondrial biogenesis were studied by Hiramitsu and collaborators [40]. Eriocitrin (eriodictyol 7-rutinoside) is present in lemon and lime [41], whose anti-oxidant and lipid-lowering activities were previously demonstrated in rats [42,43]. A zebrafish model of diet-induced obesity (DIO-zebrafish) was fed by an oral administration of eriocitrin (32 mg/kg/day for 28 days). An improved dyslipidaemia and decreased lipid droplets in the liver were evident. Moreover, eriocitrin activated mitochondrial biogenesis both in vivo and in vitro in HepG2 cells, resulting in a protective effect against the hepatic oxidative damage observed in DIO-zebrafish [40]. The robustness of this zebrafish model prompted us to employ it to prove the anti-obesity properties of two polyphenol-rich extracts from *Vitis vinifera* (grapes) and *Citrus sinensis* (orange), where we observed that these phytocomplexes were able to lower adipocyte size and number, modulating appetite-regulating hormones [44,45].

Pinostrobin is a flavonoid isolated mainly from pine (*Pinus strobus* L.), pigeon pea, Thai ginger, honey, propolis and many others. The effects of pinostrobin as neuroprotective drug in neurotoxin-induced Parkinson’s disease (PD) using zebrafish as experimental model were analyzed by Li and collaborators in 2018 [46]. The etiology of PD is not clear, even if the role of oxidative stress in the pathogenesis was demonstrated. The exposure to 1-methyl-4-phenyl-1,2,3,6-tetrahydropyridine (MPTP), a common stressor employed to mimic experimental PD, induced the loss of dopaminergic neurons and improved behavior deficiency in zebrafish. The treatment with pinostrobin showed antioxidant activity, increasing anti-oxidant enzymes such as glutathione peroxidase (GSH-Px), superoxide dismutase (SOD) and catalase (CAT), as well suppressing mitochondria-mediated neural apoptosis, via the nuclear factor erythroid 2-related factor 2 (Nrf2) pathway, thus having a potent neuroprotective effect.

In 2019, Dumitru et al. [47] investigated the effects of agathisflavone, a biflavonoid isolated from *Schinus polygamus* (Cav.) Cabrera, on memory impairment and anxiety, induced by scopolamine in zebrafish. Alzheimer’s disease (AD), a well-known neurodegenerative disorder, is characterized by the accumulation of β-amyloid deposits and neuritic plaques in brain cells, hence an augmented oxidative status. The zebrafish model proved to be useful, considering as signs of neurological impairment altered locomotion tracking patterns with modification of spontaneous alternation behavior. In this study, the acetylcholinesterase (AChE) activity, usually increased in cholinergic neurons damage, was significantly attenuated after treatment with agathisflavone. Similarly, SOD, CAT, GSH-Px activities and malondialdehyde (MDA) levels, decreased after scopolamine administration, were significantly restored after the flavonoid treatment. These data support the potential of agathisflavone in hampering brain oxidative damages, improving memory and decreasing anxiety.

Naringin was analyzed in 2019 [48] as a potential drug against alcoholic liver disease (ALD), using larvae, wild-type and transgenic adult zebrafish, expressing LFABP:GFP, all exposed to ethanol to obtain an ALD model. It was demonstrated that the administration of naringin downregulated hepatic steatosis, inducing a decrease in lipid deposition, as well as restoring LFABP expression, an index of liver functionality. Moreover, given the known close relationship between oxidative stress and lipid metabolism, ethanol exposure induced a significant increase in superoxide radical levels, whereas naringin reversed these levels, suggesting its protective effect on the liver against oxidative stress induced by ethanol. In addition, DNA damage and oxidation are usually simultaneously present, having a central role in alcoholic liver injury. In this regard, naringin also showed an important effect in inhibiting apoptosis. Therefore, naringin proved to be a powerful therapeutic drug against alcohol-induced liver injury.

Recently, an extract (TLE) of the leaves of *Curcuma longa* (turmeric) was evaluated for its antioxidant potentiality in Vero cells and in zebrafish by Kim and coworkers [49]. Turmeric has been used as a medicinal plant endowed with several biological activities, due to its content of polyphenolic compounds, among which flavonoids [49]. The TLE reduced hydrogen peroxide-induced ROS levels in Vero cells, as well as the population of cells in sub-G1, hence in apoptosis. In zebrafish exposed to the oxidant, TLE diminished cell death, ROS generation, and lipid peroxidation, proving TLE’s reliability as a promising antioxidant. Evidence on the antioxidant effect of flavonoids assessed through zebrafish is summarized in Table 1.

### 2.2. Antiangiogenic and Antitumor Effects

A fundamental role in vascular formation and development during embryonic stages is played by angiogenesis [50,51]. This process is relevant also in cancer, auto-immune and cardiovascular diseases, inflammation and infections [52,53,54,55].

The proliferation and migration of endothelial cells are part of the angiogenesis process, including in tumor growth, where the important role of novel vessels is to carry essential nutrients to the tumor tissue [56]. Therefore, pro-angiogenic molecules are clearly involved in tumor formation and development, hence all the mechanisms inhibiting angiogenesis should theoretically influence the tumor growth. In this way, the use of new drugs hampering angiogenesis could successfully treat cancers and other diseases.

One of the first molecules analyzed using zebrafish as experimental model was deguelin [57], a derivative of rotenone and part of the flavonoid family, whose potential chemopreventive activities against several types of cancers have been shown [58]. Indeed, the therapeutic efficacy of deguelin was clearly demonstrated in aerodigestive tract cancer, non-small cell lung cancer (NSCLC) and head and neck squamous cell carcinoma (HNSCC), where it inhibited hypoxia inducing factor (HIF)-1a expression at both translational and post-translational levels and suppressed vascular endothelial growth factor (VEGF), hence the angiogenic process. Deguelin inhibited HIF-1a protein synthesis, also inducing ubiquitin and proteasome mediated protein degradation, and therefore leading to a decreased VEGF production.

In 2010, Tang et al. [59] investigated the use of calycosin, an isoflavonoid isolated from *Radix Astragali*, as an anti-angiogenic agent. *Radix Astragali* is a Chinese medicinal herb commonly used for treating cardiovascular disorders and has been shown to possess angiogenic effects, but its active constituents and underlying mechanism remains unclear. *Radix Astragali* is rich in isoflavonoids and is often used either alone or in combination with other Chinese medicines in the treatment of myocarditis, heart failure, myocardial infarction, pulmonary hypertension, chronic hepatitis, diabetes and systemic lupus erythematosus [8,60]. Danggui buxue tang (DBT), a Chinese herbal concoction composed of *Radix Astragali* and *Angelica sinensis*, is commonly used for the treatment of menopausal irregularity and menstrual disorders [61,62,63]. Calycosin, thanks to its benefits upon endothelial cells, could be a very important small-molecule angiogenic agent [64], protecting human umbilical vein endothelial cell cultures (HUVECs) from hypoxia-induced barrier impairment. The promoting regeneration of cAMP levels, and so the increase in the intracellular energetic sources, is also closely related with the improving cytoskeleton remodeling. Calycosin acts on estrogen receptors and promotes angiogenesis in HUVEC cultures, either in vitro or in vivo, in a transgenic zebrafish model.

The angiogenic effects of calycosin on the subintestinal vessels (SIVs) were also analyzed in zebrafish embryos by Li et al. in 2011 [65]. The role of calycosin in the modulation of VEGF, fibroblast growth factor (FGF) and ErbB signaling pathways was demonstrated by the transcriptional profiling by deep sequencing and the quantitative real-time PCR (qPCR). Therefore, the relationships between the morphological and the genomic evidence, thereby demonstrating the role of key signaling pathways in angiogenesis, were shown.

Nobiletin (5,6,7,8,30,40-hexamethoxyflavone) was identified from the peel of citrus fruits. Lam et al., 2011 [66] investigated its anti-angiogenic role in live transgenic zebrafish embryos. The anti-angiogenic activity was demonstrated by the inhibition of the intersegmental vessels (ISVs) formation, expressing the proto-oncogene friend leukemia integration 1 (FLI1):GFP in the vasculature, along with inducing VEGF-A mRNA expression. In HUVECs, nobiletin dose-dependently inhibited endothelial cell proliferation and tube formation and in in vivo study, it induced G0/G1 phase cell cycle arrest without an increase in apoptosis, thereby showing a cytostatic effect. Moreover, anti-inflammatory activity [67], particularly for air way inflammation [68], as inhibitor of ROS production [69], anticancer [70,71,72] and against memory deterioration [73,74] are reported in the literature.

Hesperetin, naringin, neohesperidin, scutellarein, scutellarein tetramethylether and sinensetin are polymethoxylated flavonoids present in citrus fruit studied by Lam et al. in 2012 [75]. Their anti-angiogenic activity was analyzed in both HUVECs and zebrafish, where they demonstrated different degrees of effectiveness. In HUVEC cells, the angiogenesis inhibition was caused by inducing cell cycle arrest in the G0/G1 phase, whereas in zebrafish, by downregulating the mRNA expression of the angiogenesis genes flt1, kdrl, and hras. A better anti-angiogenic activity in a flavonoid with a methoxylated group at the C3- position, such as sinensetin, was shown by the SAR analysis, whereas the absence of a methoxylated group at the C8 position has lower lethal toxicity. The modification of the chemical structure of polymethoxylated flavonoids affects the antiangiogenic effects, therefore new possibilities of antiangiogenic therapies by means of chemical modifications can be hypothesized.

Quercetin is a flavonoid isolated from *Hypericum attenuatum* Choisy, whose role in the inhibition of angiogenesis was studied in vivo on transgenic zebrafish embryos and in vitro on HUVECs by Lin et al., in 2012 [76]. *H. attenuatum* Choisy is a Chinese herbal medicine commonly used for the treatment of hemorrhagic diseases. Numerous data present in the literature demonstrate that quercetin carries out anti-oxidant, anti-inflammatory and anti-tumor activities [37]. The antiangiogenic role of quercetin-4′-O-β-D-glucopyranoside (QODG) was demonstrated either in HUVECs or in zebrafish via suppressing VEGF-induced phosphorylation of VEGF receptor 2 (VEGFR2). The inhibition of VEGFR2-mediated signaling with the involvement of some key kinases such as c-Src, FAK, ERK, AKT, mTOR and S6K and the apoptosis induction inhibits angiogenesis, hence showing that QODG is a VEGFR2 kinase inhibitor, and that the anti-angiogenic activity is carried out also by VEGFR2-mediated signaling pathway. The anti-angiogenic activity of quercetin was also investigated in 2014 by Zhao et al. [77] in zebrafish embryos and HUVECs. The formation of ISVs, dorsal aorta and posterior cardinal vein was clearly inhibited by quercetin in transgenic zebrafish embryos. In HUVECs, in a concentration-dependent way, cell viability, the expression of VEGFR2 and tube formation were inhibited. Moreover, quercetin was involved in suppressing, both in vivo and in vitro, the extracellular signal-regulated kinase signaling pathway and the VEGFR2-mediated signaling pathway in endothelial cells, proving the anti-angiogenic properties of quercetin.

The antitumor and anti-angiogenic effects of *Macrothelypteris viridifrons* were analyzed by Wei et al. in 2012, along with its flavonoid quali-quantitative composition [78]. *M. viridifrons* is used in China in cancer, hydropsy, traumatic bleeding, diarrhea and rheumatism therapy, and eighteen flavonoids were identified in it. Several pieces of data are present in the literature on the antitumor activities of some *Macrothelypteris* species [79,80,81,82]. In an in vivo tumor model of mice bearing H22 hepatoma cells transplantation, the anti-cancer effects of *M. viridifrons* were investigated. The anti-angiogenic activity was analyzed showing the effects on the in vitro proliferation, migration, and tube formation of HUVECs. Moreover, transgenic zebrafish Tg (VEGFR2: GFP) and wild-type zebrafish embryos were used to evaluate in vivo the anti-angiogenic effect. *M. viridifrons* inhibited the tumor growth and the expression of VEGF and CD34, proliferation, migration and tube formation of HUVECs in vitro and also the ISVs formation in zebrafish embryos, so confirming a significant antiangiogenic effect. Therefore, *M. viridifrons* can be considered a drug with significant anti-angiogenic and antitumor effects.

Eupatilin, a bioactive flavonoid derived from *Artemisia asiatica*, has been recently studied by Lee and collaborators to deeply investigate the mechanisms underlying its well-known anticancer properties [83]. In this study, eupatilin proved to be an effective anti-proliferative agent in ovarian cancer cells, inhibiting apoptosis, blocking the cell cycle and other intracellular pathways. Moreover, eupatilin inhibited tumorigenesis in a zebrafish xenograft model, as well as suppressing both VEGF and its receptor expression, hence angiogenesis in zebrafish transgenic models. Evidence on the antiangiogenic and antitumor effects of flavonoids assessed through zebrafish is summarized in Table 2.

### 2.3. Protection from Ultraviolet (UV) Radiation

Ultraviolet (UV) radiation exposure is the most important cause of sunburn and erythema, acting also as a carcinogenic component even if only a small percentage of UVB radiation reaches the earth (around 0,5%) [84,85,86,87,88]. The continuous exposure to sunlight is also the cause of premature skin aging [89,90].

Kim et al., in 2008 [91] investigated the effects of haginin A, a metabolite of *Lespedeza cyrtobotrya*, on melanogenesis in Melan-a cells considering the extracellular signal-regulated protein kinase (ERK) and Akt/protein kinase B (PKB) pathway activation, using the zebrafish as experimental model. The *Lespedeza* species (Leguminosae) are known to produce a number of unique isoflavonoids and also have antioxidant activity [89]. A down-regulation of melanin synthesis in Melan-a cells was induced by haginin A, causing hypopigmentary effects. This effect was also seen in zebrafish and was probably due to decreased tyrosinase production, as well as the hindering of the signaling pathways responsible for its regulation.

In 2009 Wang et al. [92] developed a whole-organism zebrafish model for screening new compounds for protection against UVB radiation to reveal phenotypic changes of zebrafish embryos. The tolerance to UVB and the embryonic stages are in close relationship [93], since already in the embryos a photorepair mechanism is present after UVB treatment. Imbalances between UVB and photorepair activity cause cell injuries, but the embryos die if the photorepair activity is unable to bear the extremely high intensity of UVB. Three different exposure methods to administer different UVB doses at different time were used, showing that no or very temporary damage was caused by low doses of UVB while embryo death occurred after high doses. The most evident consequences after exposure to UVB were fin, especially pelvic fin, malformation (reduced and/or absent fin). Experiments of “prevention” and “treatment” by the use of green tea extract and/or epigallocatechin (EGCG) to test this whole-organism model were carried out. The effects of green tea extract and EGCG on the morphological modifications and the fins development (especially pelvic fins) after UVB exposure were assessed using the Kaplan–Meier analysis, log-rank test and Cox proportional hazards regression. Therefore, the zebrafish is effective as a whole-organism model to screen new compounds such as small chemicals for sun protection activity.

Yang et al. in 2012 [94] demonstrated the role of quercitrin-3-O-rhamnoside (QR) in the protection against ultraviolet B-induced cell death both in vitro and in zebrafish. UVB-induced formation of the intracellular ROS plays a role in the modulation of apoptosis [95,96]. The suppression of ROS levels and their generation was shown after treatment with QR, with an important effect also in preventing UVB-induced cell death. It was demonstrated that the QR has a cytoprotective effect on UVB-induced cell injuries in HaCaT human keratinocytes, especially on those generated by the exposure of these cells to UVB radiation. Therefore, QR reduced UVB-induced cell death and apoptosis in HaCaT cells and reduced UVB-induced ROS generation and cell death in live zebrafish.

Antioxidant and skin whitening properties were demonstrated in *Alnus cordata* by Smeriglio et al., in 2019 [97]. Analyzing the phenolic composition, a significant presence of flavanones (26.74%), such as epicatechin, orientin, isovitexin, naringenin and eriodictyol was shown, therefore confirming that zebrafish is a useful model for the in vivo analysis of anti-melanogenesis agents. Evidence on the protective effects of flavonoids against UVB radiation assessed through zebrafish is summarized in Table 3.

### 2.4. Anti-Inflammatory Effects

Chen et al. in 2013 evaluated the anti-inflammatory effects of flavonoids and particularly chalcone (1,3-diphenyl-2-propen-1-one) and its derivatives in a zebrafish model [98]. Chalcone is a phenolic compound present in vegetables, that, with its analogues, carries out antioxidant, anti-inflammatory, antiparasitic and antitumor activities [99,100]. In the acute inflammation process the presence of pro-inflammatory mediators, such as myeloperoxidase (Mpx), NF-κB and tumor necrosis factor alpha (TNF-α), as well as neutrophils is very significant [101]. The transparency of zebrafish embryos allows a precise, noninvasive and dynamic in vivo observation. Therefore, a transgenic zebrafish line Tg (Mpx:GFP) under the control of neutrophil-specific Mpx promoter was used [102] to count the neutrophils’ number, to check their migration and to monitor the neutrophils’ migration activity. The Mpx expression was investigated by histochemistry and the protein levels of three pro-inflammatory factors (Mpx, NFκB, and TNFα) after treatment with chalcones were analyzed. The treatment with a chalcone derivative influenced the neutrophil migration and also Mpx enzymatic activity. The protein expression levels of pro-inflammatory factors, such as Mpx, NF-κB, and TNF-α, clearly showed the anti-inflammatory effects of synthesized chalcone.

The effects of some flavones on the apoptosis of neutrophils with their anti-inflammatory effects through down-regulation of Mcl-1 via a proteasomal dependent pathway were investigated by Lucas et al. in 2013 [103]. Neutrophil apoptosis followed by a non-phlogistic clearance by phagocytes has an evident anti-inflammatory effect; therefore, the flavones apigenin, luteolin, and wogonin were used to induce the in vitro neutrophil apoptosis and to obtain an in vivo anti-inflammatory effect. All the three flavones induced time- and concentration-dependent neutrophil apoptosis showed by morphological observation through light microscopy and flow cytometry and this induction of apoptosis was caspase-dependent. Moreover, in a zebrafish model of sterile tissue injury, wogonin exerted an anti-inflammatory effect due to the in vivo neutrophil apoptosis, blocked by caspase inhibition. Therefore, the abovementioned flavones induce neutrophil apoptosis, hence having a significant anti-inflammatory effect.

In 2018, Rishitha et al. used a zebrafish model in a study regarding the treatment of neurodegenerative disorders by the administration of solid lipid nanoparticles (SLN) of quercetin [104]. Particularly, cognitive impairment was induced in zebrafish by pentylenetetrazole (PTZ). The anti-inflammatory role of quercetin was demonstrated by the decrease in lipid peroxidation together with an antioxidant role, raising the reduced glutathione. Moreover, the neurotransmitter regulation was carried out by a reduction of AChE activity, with a potential neuroprotective and cognitive improvement. Therefore, using the zebrafish model quercetin SLN are future nanomedicine that could be useful in different neurodegenerative diseases, such as Parkinson’s and Alzheimer’s disorders.

We recently employed a zebrafish model to assess the anti-inflammatory effect of a flavonoid-rich extract of orange (OJe), after *Vibrio anguillarum* infection to induce bacterial enteritis. The pretreatment with OJe helped reduce the outcomes of the induced enteritis both at microscopic and molecular levels. In particular, OJe hindered the rise of pro-inflammatory cytokines, such as IL-1β, IL-6 and TNF-α, indicating its value for the prevention of enteritis and the amelioration of the consequent inflammatory status [105]. Evidence on the anti-inflammatory effect of flavonoids assessed through zebrafish is summarized in Table 4.

### 2.5. Toxicity of Flavonoids Employing the Zebrafish as Model

In 2016, Bugel and collaborators used a zebrafish embryo-larval bioassay as an alternative screening platform for the determination of the developmental toxicity and bioactivity of 24 flavonoids and flavonoid-like chemicals in vivo [106]. The early life exposure effects on morphological outcomes, behavior and gene expression for all compounds and for effects on biomarkers’ transcripts indicative of the aryl hydrocarbon receptor (AHR; *cyp1a*) and estrogen receptors (ER; *cyp19a1b*, *esr1*, *lhb*, *vtg*) were evaluated. Among the 24 flavonoids tested, 15 were developmentally bioactive and elicited adverse effects on morphology and/or behavior. In addition, the evaluation of biomarker gene expression showed chemical and gene-specific outcomes resulting in complex estrogenic and anti-estrogenic effects. This study demonstrated that the developmental toxicity was not ER- or GPER (G protein-coupled estrogen receptor)-dependent, indicating alternative modes of action, thus confirming the utility of the integrative, multidimensional and translational zebrafish platform for examining the developmental toxicity of xenobiotics.

## 3. Conclusions

**Figure 4:**
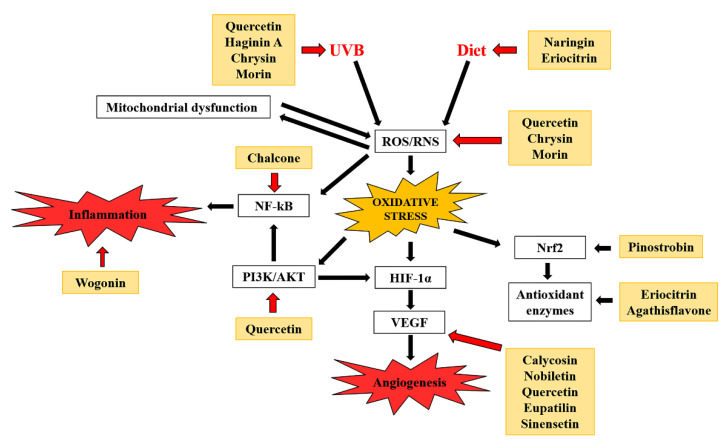
Molecular mechanisms underlying flavonoid action against oxidative stress disorders. Black and red arrows indicate induction and inhibition, respectively.

In the last few decades, zebrafish has been extensively considered as the real new experimental model, used in numerous fields of developmental biology and the medical sciences for many different reasons: (i) optimal small size for microscopic examination; (ii) potential for genetic manipulation and the transparency of their embryos; and (iii) gene expression similarity with human species. Moreover, the important economic advantage with respect to other species such as mice, commonly used for experiments, is one of the main strengths of this model. Several natural and synthetic drugs were screened and optimized on zebrafish, underlining the potential of this experimental model to study oxidative stress-linked disorders. Here, we reported that flavonoids were able to increase oxidative defenses in zebrafish against a multitude of several stressors (i.e., heavy metals, UV radiations, high-fat diets, neurotoxins), interfering with angiogenesis at different levels, as well as hampering the inflammatory status (Figure 4). Therefore, zebrafish can be helpful to evaluate new therapeutic strategies for oxidative stress-linked disorders.

## Figures and Tables

**Table 1:** Studies employing zebrafish to assess the antioxidant activity of flavonoids.

Ref.	Flavonoid	Model	Effects
[36]	quercetin 3-*O*-methyl ether	zebrafish exposed to 5μM Cu^2+^	protective role against oxidative damage
[39]	15 flavonoids	UVB-exposed embryos zebrafish	reduction of ROS
[40]	eriocitrin	DIO-zebrafish	improved dyslipidaemia and decreased lipid droplets in the liver; increased mRNA of mitochondria transcription factor, nuclear respiratory factor 1, cytochrome c oxidase subunit 4 and ATP synthase
[46]	pinostrobin	MPTP-exposed zebrafish	increase in anti-oxidant enzymes such as GSH-Px, SOD and CAT; suppression of mitochondria-mediated neural apoptosis by Nrf2 pathway
[47]	agathisflavone	Scopolamine-treated zebrafish	restoration of SOD, CAT, GSH-Px activities and MDA levels, decreased after scopolamine administration
[48]	naringin	Ethanol-exposed zebrafish larvae	reduction of lipid accumulation and superoxide radical levels

UVB: ultraviolet B; DIO: diet-induced obese; MPTP: 1-methyl-4-phenyl-1,2,3,6-tetrahydropyridine; ROS: reactive oxygen species; ATP: adenosine triphosphate; GSH-Px: glutathione peroxidase; SOD: superoxide dismutase; CAT: catalase; Nrf2: nuclear factor erythroid 2-related factor 2; MDA: malondialdehyde.

**Table 2:** Antiangiogenic and antitumor effects of flavonoids in zebrafish.

Ref.	Flavonoid	Model	Effects
[65]	calycosin	zebrafish embryos	modulation of VEGF, FGF and ErbB signaling pathways
[66]	nobiletin	transgenic zebrafish embryos	inhibited formation of ISVs; induced G0/G1 phase accumulation in FLI1-positive endothelial cells; induced VEGF-A mRNA expression
[75]	7 polymethoxylated flavonoids	zebrafish	downregulation of the mRNA expressions of angiogenesis genes flt1, kdrl, and hras
[76]	quercetin-4′-O-β-D-glucopyranoside	transgenic zebrafish	suppressed VEGF-induced phosphorylation of VEGFR2 through the involvement of c-Src, FAK, ERK, AKT, mTOR and S6K
[77]	quercetin	transgenic zebrafish embryos	VEGFR2 inhibition
[77]	quercetin	transgenic zebrafish embryos	inhibited the formation of ISVs, dorsal aorta and posterior cardinal vein
[80]	calycosin	transgenic zebrafish	promotion of angiogenesis through estrogen receptor and MAPK
[83]	eupatilin	zebrafish xenograft model	inhibited tumorigenesis; suppressed both VEGF and its receptor expression

VEGF: vascular endothelial growth factor; FGF: fibroblast growth factor; ISV: intersegmental vessel; FLI1: friend leukemia integration 1; FAK: focal adhesion kinase; ERK: extracellular signal-regulated kinase; AKT: protein kinase B; mTOR: mammalian target of rapamycin.

**Table 3:** Studies assessing flavonoid effects against UVB-induced toxicity in zebrafish models.

Ref.	Flavonoid	Model	Effects
[91]	haginin A	UVB-exposed zebrafish	decreased tyrosinase production
[94]	quercitrin-3-O-rhamnoside	UVB-exposed zebrafish	reduced UVB-induced ROS generation and cell death

**Table 4:** Studies employing zebrafish to assess the anti-inflammatory activity of flavonoids.

Ref.	Flavonoid	Model	Effects
[98]	chalcone	transgenic zebrafish	affected wound-induced neutrophil recruitment, Mpx enzymatic activity, protein expression levels of Mpx, NF-κB, and TNF-α
[103]	wogonin	zebrafish model of sterile tissue injury	neutrophil apoptosis blocked by caspase inhibition
[104]	quercetin	pentylenetetrazole-treated zebrafish	decrease in lipid peroxidation together with an antioxidant role, and reduced glutathione

Mpx: myeloperoxidase; NF-κB: nuclear factor kappa B; TNF-α: tumor necrosis factor alpha.
